# Resource Allocation during an Influenza Pandemic

**DOI:** 10.3201/eid1410.080371

**Published:** 2008-10

**Authors:** Nick F. Phin, Lindsey Davies

**Affiliations:** Health Protection Agency Centre for Infections, London, UK (N.F. Phin); Department of Health, London (L. Davies)

**Keywords:** Pandemic influenza, resource allocation, prioritization, letter

## To the Editor

Considerable progress has been made in the United Kingdom to prepare for an influenza pandemic. After public consultation, an updated national framework (*1*) was recently published, along with new guidance on ethics (*2*), surge capacity, and clinical prioritization (*3*).

As Paranthaman et al. pointed out (*4*), difficult ethical choices must be made during a pandemic. Therefore, the UK Committee on Ethical Aspects of Pandemic Influenza published the ethical framework (*2*) designed to assist with and support the ethical aspects of policy and clinical decision making during and after an influenza pandemic. The fundamental principle underpinning the ethical framework is equal concern and respect, and it is expected that this principle, supported by 7 others listed in the guidance, will be used by clinicians, managers, and healthcare planners to develop policies on clinical issues for use during a pandemic. It is recognized and acknowledged within the document that the weight of a given principle will vary according to the circumstance.

Equally relevant is the interim guidance on surge capacity and prioritization in health services (*3*), which sets out a framework for the health service response in the United Kingdom during a pandemic and which advocates the wider use of the clinical triage criteria described for critical care by Christian et al (*5*). The proposed use of clinical triage at the primary care/secondary care interface starts to address the issue raised by Paranthaman et al. of who should be admitted to a hospital. This guidance expands early UK guidelines on the management of influenza-like illness during a pandemic.

We agree with Paranthaman et al. that early surveillance data are needed to rapidly inform clinical care guidelines in a pandemic. Therefore, efforts are ongoing to increase the resilience of health surveillance data gathering systems in the United Kingdom and to develop clinical systems for specific use during a pandemic. At the onset of a pandemic, it is intended that data will be gathered on the first few hundred patients by using a modification of the Web-based avian influenza management system of the Health Protection Agency. These data will provide important virologic and epidemiologic information to characterize the pandemic virus and inform modeling assumptions to validate “now casting” or real-time mathematical models (*6*) being developed in the United Kingdom and Europe to estimate the likely spread and impact of the pandemic. Furthermore, pilot projects are in preparation to develop clinical data collection systems in secondary care to assess treatments and outcomes during a pandemic.

In conclusion, contingency decisions outside normal patient pathways will be needed; the UK guidance, based on current knowledge and understanding, will help clinicians make difficult decisions on patient prioritization, plan surge capacity, build resilience into existing surveillance systems, and develop new systems that seek to inform the best use of resources to deliver optimal clinical care during an influenza pandemic. These decisions will be revised and modified to reflect new developments in the science.
