# Supplementary material for: Resource Allocation during an Influenza Pandemic
Source: Emerg Infect Dis. 2008 Oct;14(10):1676–7. doi: 10.3201/eid1410.080371 (PMC2609899; doi:10.3201/eid1410.080371)
Supplement: Related texts

# Reply: In Response

**Authors:** Karthikeyan Paranthaman, Christopher P. Conlon, Claire Parker, Noel McCarthy

**Affiliations:** Public Health Department, Oxford, UK (K. Paranthaman); John Radcliffe Hospital, Oxford (C.P. Conlon); Jericho Health Centre, Oxford (C. Parker); Thames Valley Health Protection Unit, Oxford (N. McCarthy)

We are grateful to Phin and Davies for providing an update on the pandemic flu planning situation in the United Kingdom (*1*) after publication of our letter (*2*). We agree with their emphasis that pandemic planning in the United Kindgom is in many regards well developed in comparison with other countries. Many of the updates that they describe provide useful contributions but the final version of UK ethical guidance is more a general statement of principles than a practical guide. Unfortunately, it has pulled back from some of the more definite statements in the earlier consultation documents available at the time of our letter.

An important need for practical guidance for frontline and managerial staff on the ethical aspects of pandemic response remains as a gap in the United Kingdom and elsewhere. For critical care, a useful recent contribution is the report produced by the Task Force on Mass Critical Care, especially the framework for allocation of scarce resources in mass critical care (*3*). The shift away from pandemic influenza–specific criteria to a more generic framework for resource allocation that can compare patients with pandemic influenza and those with other usual noninfluenza conditions is welcome.

Similar practical approaches outside the critical care area are important priorities for work in pandemic preparedness. In addition, systems must be in place for rapid modification and communication of the criteria for referral and admission, taking into account the severity of the pandemic and availability of healthcare resources. Healthcare services must not only be transparent and fair in this process, they must also be seen by the public as being so. Engagement with the public on values and principles underlying resource allocation would be of paramount importance during a pandemic.

## References

[1] Phin NF, Davies L. Resource allocation during an influenza pandemic [letter]. Emerg Infect Dis. 2008;14:1676. 10.3201/eid1410.08037118826850PMC2609899

[2] Paranthaman K, Conlon CP, Parker C, McCarthy N. Resource allocation during an influenza pandemic. Emerg Infect Dis. 2008;14:520–2. 10.3201/eid1403.07127518325284PMC2570815

[3] Devereaux AV, Dichter JR, Christian MD, Dubler NN, Sandrock CE, Hick JL, Definitive care for the critically ill during a disaster: a framework for allocation of scarce resources in mass critical care. Task Force for Mass Critical Care Summit Meeting, 2007 Jan 26–27. Chicago. Chest. 2008:51S–66S [cited 2008 Jun 12]. Available from http://www.chestjournal.org/cgi/content/abstract/133/5_suppl/51S10.1378/chest.07-269318460506
